# Fruit and Agro-Industrial Waste Extracts as Potential Antimicrobials in Meat Products: A Brief Review

**DOI:** 10.3390/foods10071469

**Published:** 2021-06-25

**Authors:** Leticia Aline Gonçalves, José M. Lorenzo, Marco Antonio Trindade

**Affiliations:** 1 Faculdade de Zootecnia e Engenharia de Alimentos, Universidade de São Paulo, Av. Duque de Caxias Norte, Pirassununga 13635-90, São Paulo, Brazil; trindadema@usp.br; 2 Área de Tecnología de los Alimentos, Facultad de Ciencias de Ourense, Universidad de Vigo, 32004 Ourense, Spain; jmlorenzo@ceteca.net; 3 Centro Tecnológico de la Carne de Galicia, Rúa Galicia N-4, Parque Tecnológico de Galicia, San Cibrao das Viñas, 32900 Ourense, Spain

**Keywords:** *Opuntia ficus-indica*, *Myrciaria cauliflora*, *Vitis* sp., microbiological stability, sensory properties, cleaner label

## Abstract

The use of antimicrobials in meat products is essential for maintaining microbiological stability. The reformulation by substituting synthetic additives for natural ones is an alternative to provide cleaner label products. Therefore, this work performed a literature search about extracts from fruits and agro-industrial waste with antimicrobial activity that can be applied in meat products. Jabuticaba waste extracts are excellent sources of anthocyanins with antimicrobial and pigmentation potential, capable of being applied in meat products such as fresh sausage, without compromising sensory attributes. Residue from grapes is rich in antimicrobial phytochemicals, mainly catechins, epicatechins, gallic acid and procyanidins. Extracts from different grape by-products and cultivars showed inhibition of *Staphylococcus aureus*, *Listeria monocytogenes*, *Pseudomonas aeruginosa*, *Escherichia coli* O157: H7 and other bacterial strains. Antimicrobial effects against *L. monocytogenes*, *Bacillus cereus*, *S. aureus* and *E. coli* O157: H7 were identified in *Opuntia* extracts. In addition, its application in hamburgers reduced (*p* < 0.05) aerobic mesophilic bacteria, *Enterobacteriaceae* and *Pseudomonas* sp. counts, and at a concentration of 2.5%, improved the microbiological stability of salami without causing sensory and texture changes. These data reinforce the possibility of substituting synthetic preservatives for natural versions, a growing trend that requires researching effective concentrations to maintain the sensory and technological properties.

## 1. Introduction

Since antiquity, even without being aware of the proliferation of microorganisms, when observing the high perishability of meat and the need for its immediate consumption, man began to use techniques of physical and chemical changes capable of delaying spoilage and improving the flavor of this and other food classes, which allowed the significant extension of the availability period of certain foods. One of the oldest forms of meat processing is the manufacture of by-products from the processing of meat pieces, which started around 1500 BC in the Mediterranean region, whose climate was favorable for the maturation of products, when several procedures that resulted in the reduction in water activity and consequently the prolongation of their shelf life, such as desiccation, drying, curing, smoking, salting and/or mixture of aromatic herbs, were also applied [1,2].

As they are nutritionally rich foods with a large amount of available water in their composition, meats become susceptible to contamination by pathogenic and spoiling microorganisms. In order to overcome this problem and offer safe meat products to consumers, it is necessary to adopt measures for their conservation, such as good manufacturing practices, use of low temperatures during storage, heat treatment and use of additives [3].

Processing has the purpose of extending the shelf life of meat, adding value to deboning by-products, which are generally not marketed in the fresh form, in addition to generating a wide variety of differentiated products in terms of color, flavor, aroma and texture [4]. Due to the low cost and easy preparation, a considerable part of the population developed the habit of regularly consuming meat derivatives such as sausages, bologna and hamburgers, contributing to a significant expansion of the meat products market [5,6]. 

The quality of meat derivatives is directly related to the origin of raw materials and ingredients and to the sanitary conditions of the manufacturing process. Products are classified according to the types of meat used, fat content, offal or edible by-products from slaughter, and may or may not be added with condiments and additives permitted by legislation [1]. In the meat production process, meat comminution increases the contact surface area, favoring microbial contamination and proliferation [7]. Therefore, the incorporation of preservatives in processing can considerably contribute to the maintenance of quality and safety characteristics during shelf life [8]. The use of these substances in meat products is regulated in Brazil by RDC No. 272/2019, which regulates the use of food additives for each class of meat derivative, their conditions of use and maximum limits [9].

For a long time, the food industry has incorporated various ingredients into formulations that do not have the function of nourishing, but rather have a technological purpose, while they can also make the food more attractive to consumers. These ingredients are called food additives and are classified according to their technological function [10]. The class of preservatives is one of them, the main purpose of which is to reduce the effect of spoilage caused by the multiplication of microorganisms or chemical reactions during the storage period [11,12]. Synthetic substances that have their use approved within an acceptable daily intake limit, such as nitrite and sodium nitrate, preservatives most commonly used in the production of meat derivatives, are also used, which in addition to their antimicrobial capacity, particularly for the control and prevention of the growth of anaerobic bacteria, especially *Clostridium botulinum*, also promote a protective effect against lipid oxidation and act in the development and fixation of the pink color and flavor characteristics of cured meat products [2,10,12].

The application of sodium nitrite in the production of cured meats allows obtaining differentiated products with regard to color and flavor, safe and stable during storage. However, a discussion that started around the 1970s showed the great risk to human health from the generation of a class of substances considered potentially carcinogenic, the nitrosamines, when high nitrite concentrations are exposed to high temperature conditions, as usually occurs in the manufacture of cured meat products, and since then, its use has been considered increasingly controversial [2,13].

Although they are substances that significantly contribute to the conservation of products and have their use officially regulated, there are indications of negative health implications associated with the excessive consumption of these and other synthetic additives, such as carcinogenic effects and generation of toxic and mutagenic compounds, and, consequently, the maximum acceptable limits of their use have been gradually changed or prohibited in several countries [14,15].

Diet is one of the important factors that affect the well-being and health of human beings, and today, there is great concern among consumers about the correlation between eating habits and health problems [16,17]. The reformulation of meat products through the substitution of ingredients, such as sodium nitrite, is an alternative to provide these products with a “cleaner label” in order to reduce the negative consumer perception about the excessive use of synthetic additives and their carcinogenic potential, decreasing the association between consumption of meat products and possible health problems [16,17]. For these reasons, many studies have been conducted in order to substitute synthetic antimicrobials for natural versions.

## 2. Phenolic Compounds

Phenolic compounds are phytochemicals with functions in pigmentation, astringency, protection against ultraviolet rays and antioxidant activity, being widely found in natural sources such as fruits, teas, spices, wine and honey [18]. These compounds have received much attention in recent decades due to evidence related to positive health effects, such as anti-inflammatory, antimicrobial, antithrombotic, vasodilatory and cardioprotective activity, contributing to the improvement in metabolic markers associated with diabetes, hypertension and obesity [19,20].

Phenolic molecules are structurally characterized by the presence of at least one aromatic ring containing one or more hydroxyl radicals, the main groups being phenolic acids, flavonoids and polyphenols, whose main source is fruits [18]. In addition, recent studies have reported antioxidant and antimicrobial effects of phenolic compounds, indicating that their chemical nature, especially the presence of hydroxyl groups in the molecule, may be associated with inhibitory mechanisms through interaction with the cytoplasmic membrane, cell wall and nucleic acids of bacteria, impairing vital functions such as protein synthesis and DNA transport or replication [21,22].

The generation of large amounts of waste from the processing of fruits and vegetables is one of the main challenges that the food industry has faced due to the need for large investment by companies to properly treat and dispose of this type of material in order to cause minimal negative impacts on the environment [14]. These agro-industrial waste products from fruits are rich in phenolic compounds and other bioactive substances that can add antioxidant and antimicrobial properties to foods and provide health benefits [23]. Thereby, the use of this raw material as a natural substitute for synthetic additives can be a great alternative, because in addition to providing compounds with functional properties, it reduces the environmental impact caused by the disposal of a significant part of the fruit, such as seeds and peels that are generally not used by the industry [24].

Thus, this review searched the scientific literature for reports of extracts obtained from fruits or their agro-industrial waste rich in antimicrobial bioactive compounds and that have potential applications in meat products, being able to maintain microbiological stability and safety during storage. More specifically, this review focused on the natural extracts obtained from jabuticaba, grape and prickly pear.

## 3. Fruit Extracts with Potential Application in Meat Products

### 3.1. Jabuticaba (Myrciaria cauliflora)

Jabuticaba (*Myrciaria cauliflora*), belonging to the *Myrtaceae* family, is a fruit tree native to Brazil, whose cultivation extends throughout the national territory, with greater productivity in the Southeastern region. From the nutritional point of view, jabuticaba varieties are considered excellent sources of dietary fibers, carbohydrates, vitamins and minerals such as iron, calcium and phosphorus, arousing great interest for its considerable amount of phenolic compounds with antioxidant and antimicrobial potential [25,26]. Among these compounds, anthocyanins and flavonoids are mainly concentrated in fruit peel, being the main components responsible for the development of its characteristic dark color. The anthocyanin content, of approximately 315 mg per 100 g of jabuticaba, is considered high compared to other fruits, demonstrating great potential as a substitute for synthetic dyes in several food classes, in addition to the benefits for the conservation of these products [8,27].

The most attractive source for obtaining these natural pigments from jabuticaba, rich in antioxidant and antimicrobial bioactive compounds, is the use of residues from jelly- and liquor-processing industries, since peels and seeds represent approximately 50% of the fruit and are in general discarded by the industry [28,29].

Baldin et al. [8] studied the application of microencapsulated jabuticaba extract in fresh sausage, evaluating the antimicrobial and antioxidant potential of this natural dye. Firstly, an in vitro experiment was carried out, which demonstrated an inhibitory effect on Gram-positive and Gram-negative bacteria, showing its antimicrobial potential. The minimum inhibitory concentration (MIC) results for the microencapsulated extract were 18.75 g/L (~2%) for both *Staphylococcus aureus* ATCC 25923 and *Escherichia coli* ATCC 25922. These results can be attributed to the high concentration of phenolic compounds (anthocyanins) in fruit peel, which are mainly responsible for the antimicrobial activity in this case [30].

When applying microencapsulated jabuticaba extract in fresh sausage, Baldin et al. [8] observed a reduction in the counts of mesophilic bacteria and of thermotolerant coliforms in treatments with 2 and 4% extract on the first and fifteenth days of cold storage. For *S. aureus*, treatments with 2 and 4% microencapsulated extract also showed lower counts when compared to the control (without addition of extract or dye) and with the treatment with added cochineal carmine dye. The addition of 4% extract caused the elimination of *S. aureus* on the last day of storage (15 days). In the total count of aerobic psychrotrophic microorganisms, a reduction of 1 log cycle was observed at the end of the fourth day of storage of treatments with additions of 2 and 4% of jabuticaba extract; however, from the eighth day, all treatments tested exceeded the limit of 10^7^ CFU/g recommended by the International Commission on Microbiological Specifications for Foods (ICMSF) [31], indicating spoilage that can lead to sensory loss in the attributes of odor, color and taste. The count of lactic bacteria increased from 4 log CFU/g at time zero (beginning of storage) to 6 log CFU/g at the end of storage in all treatments, not exceeding the limit of 10^7^ CFU/g established by ICMSF [31]. *Salmonella* sp. tested negative in 25 g for all treatments, being in accordance with Brazilian legislation [32].

Thus, the study recommended the addition of 2% of microencapsulated jabuticaba extract in fresh sausage, as it did not compromise the sensory attributes evaluated, except for the purplish color, which was slightly less accepted because it is not characteristic of the product. The aforementioned extract can be considered a good alternative for the production of cleaner label meat products as it satisfies the demand for the use of natural pigments with antimicrobial capacity and low cost, taking advantage of residues from the jabuticaba processing and with the appeal of health benefits.

### 3.2. Grape (Vitis sp.)

Grape (*Vitis* sp.) is one of the fruits most cultivated around the world, occupying an area of 7.5 million hectares of vineyards, with emphasis on the production of species *Vitis vinifera* in most countries [33,34]. In Brazil, the most commonly found cultivars are *Vitis labrusca*, *Vitis bourquina*, *Vitis vinifera* and several interspecific hybrids, occupying an area of 78 thousand hectares from the extreme south of the country to near the equator, showing characteristic poles of temperate, subtropical and tropical climates due to its expressive environmental diversity [35]. Brazilian grape production reached 1.5 million tons per year in 2018, with 50% destined for processing—wine making (42% table wines and 7% fine wines), juices (49%) and other derivatives (2%)—and the other half of the national volume marketed as grapes for fresh consumption [35].

The generation of waste from the processing of this high volume of grapes is significant, and may correspond to 30% of the fruit when used for the production of wines, for example, consisting of by-products such as pomace, peels and seeds [33,36]. This waste is considered a source of phenolic compounds with antioxidant and antimicrobial effects, mainly catechins, epicatechins, gallic acid and procyanidins [36,37,38].

Martin et al. [24] evaluated the in vitro antimicrobial capacity of ethanolic grape extracts from lyophilized seeds or agro-industrial waste. For the extract obtained from lyophilized pomace of “Pinot Noir” (*Vitis vinifera*) cultivar, the authors found MIC against Gram-positive *S. aureus* ATCC 25923 and *Listeria monocytogenes* ATCC 7644 pathogens of 6.25 and 12.5 g/L, respectively. “Petit Verdot” (*Vitis vinifera*) seeds ethanolic extract presented an MIC of 6.25 g/L for *L. monocytogenes* and 1.56 g/L for *S. aureus* (Table 1).

Adámez et al. [37] estimated the in vitro antibacterial activity of aqueous grape seed extract (*Vitis vinifera* L.), Tempranillo cultivar, obtained after wine manufacture, and reported efficiency in inhibiting Gram-positive 976 *S. aureus subsp. aureus* and 910 *Listeria innocuaI* (Table 1). Similar results were obtained by Baydar et al. [34] in seeds from “Hasandede”, “Emir” and “Kalecik” cultivars (all *Vitis vinifera* L. species), which demonstrated a relationship between increased extract concentration and reduced growth of 15 bacterial strains, including the pathogens *E. coli* O157: H7 KUEN 1461, *Aeromonas hydrophila* ATCC 7965 and *S. aureus* Cowan 1. Additionally, extracts at concentrations of 0.5 and 1% showed a bacteriostatic effect on *E. coli*, while concentrations of 2.5 and 5% provided bactericidal activity.

Despite the good in vitro results, data on the incorporation of this class of extract to guarantee microbiological stability in meat products were not found in thescientific literature. Carpes et al. [39] obtained lyophilized hydroethanolic (GPWL: grape pomace wine lyophilized) and microencapsulated (GPWM: grape pomace wine microencapsulated) extracts made with grape pomace from the processing of *Vitis labrusca* L. Bordeaux varieties and applied them to chicken pate in order to evaluate the effects of the addition of natural compounds on oxidative stability compared to negative control treatment (T1; no antioxidant added) and with the use of 300 ppm of the synthetic antioxidant sodium erythorbate (T2). 

The study reported satisfactory results for the inhibition of lipid oxidation in treatments with 3000 ppm of GPWL (T3) and GPWM (T4) extracts, analyzed by the index of substances reactive to 2-thiobarbituric acid (TBARs) during 42 days of cold storage (4 °C). At the end of the storage period, all treatments showed a significant difference from each other (*p* < 0.05) in relation to the TBARS assay, with the best results being observed for T3, followed by T4, T2 and T1, respectively. All treatments, except for T1, had results below 3 mg of malondialdehyde/kg of sample, a value considered the limit for the meat product to be considered adequate and safe for consumption according to some authors [40,41]. Both GPWL and GPWM demonstrated an effective reduction in lipid oxidation when compared to treatment elaborated with commercial synthetic antioxidant, an activity that can be attributed mainly to the presence of phenolic compounds such as gallic, caffeic, vanillic, ferulic and coumaric acids and trans-resveratrol [39].

Thus, it appears that the extracts obtained from grape processing waste can be considered an interesting and innovative strategy for the incorporation of bioactive compounds in meat products with the substitution of synthetic preservatives for natural ones, since studies have shown their efficiency in inhibiting the growth of microorganisms related to outbreaks of foodborne diseases, such as *Listeria*, *E. coli* and *S. aureus*. However, studies on the in vivo influence on the sensory characteristics and antimicrobial action of meat products are necessary, since in general, studies have essentially evaluated the antioxidant activity.

### 3.3. Prickly Pear (Opuntia ficus-indica)

*Opuntia ficus-indica*, popularly known as prickly pear, is the fruit of a cactus species (*Cactaceae* family) native to tropical and subtropical regions of the Americas and currently also being cultivated in Europe, Africa and Australia, with approximately 300 known varieties [42]. The literature contains plenty of information about the chemical composition of its pulp, seeds and peel, as well as some properties of interest for the pharmaceutical and food industry, because it is a natural source of bioactive compounds.

Brazil has the largest *Opuntia ficus-indica* cultivation system in South America, with a planting area of 500,000 hectares located mainly in the Northeastern region and recently extended to other regions. Cultivation is performed in general by small producers and a large part of the production is destined for animal fodder, when it is called forage palm or cattle palm [43]. In the region of Valinhos, state of São Paulo, production is destined for the generation of fruit for fresh consumption, aimed at export to Europe and the domestic market [44]. In 2017, around 18.01 tons of the fruit were sold at “Companhia de Entrepostos e Armazéns Gerais de São Paulo” (CEAGESP), ranking 326th among products sold by the company [45].

In recent years, there has been a remarkable interest from the scientific community regarding the regular consumption of the genus *Opuntia* and its positive correlation with the treatment and prevention of chronic diseases related to oxidative stress [46,47]. Benefits such as reduction in triglycerides and total cholesterol in the bloodstream [48], antiulcerogenic activity [49], improved platelet aggregation [50] and reduced renal dysfunction [51,52] are some of the pieces of clinical and/or experimental evidence associated with the consumption of fig varieties. Other authors have found that extracts from the fruit and its peel and seeds have appreciable amounts of unsaturated fatty acids [53], with antioxidant activity [54,55], anticancer effects [56] and cardioprotective, antithrombotic, anti-inflammatory, antiarrhythmic, hypolipidemic and anti-hyperglycemic activities [57,58].

Seo et al. [59] identified the antimicrobial effects of *Opuntia ficus-indica* extract on two important pathogens related to foodborne diseases, *L. monocytogenes* and *E. coli* O157: H7, suggesting that the extract can be used as a natural preservative in food products. Zito et al. [60] detected the presence of eleven substances with antimicrobial activities in peels, seeds and pulps of the yellow (Surfarina) and red (Sanguigna) fruit varieties. Of these, major components were carvacrol, limonene, squalene and hexadecanoic acid, which in addition to their antimicrobial capacity are also antioxidants.

Parafati et al. [61] applied aqueous pulp extract of the purple and red *Opuntia ficus-indica* varieties in bovine hamburgers, testing the direct addition of the extract and the version encapsulated in sodium alginate. Microbiological analyses conducted after 8 days of cold storage (4 °C) showed the preservative effect in hamburgers with added prickly pear extract, which significantly reduced (*p* < 0.05) the count of mesophilic bacteria, *Enterobacteriaceae* and *Pseudomonas* sp., when compared to control sample with the addition of sterile distilled water. The authors concluded that the application of the extract, encapsulated or not, is an effective method for conservation of bovine hamburgers. However, studies are necessary to verify the influence of this component on the sensory and technological properties of products.

The antibacterial activity of the hydroethanolic extract obtained from the whole *Opuntia stricta* fruit, another species of the genus *Opuntia*, was quantified by determining the MIC and the minimum bactericidal concentration (MBC) by Kharrat et al. [62]. The results in Table 2 show that the extract from the red variety of *Opuntia stricta* showed high antibacterial activity, with an MIC and MBC less than or equal to sodium nitrite, which is the synthetic preservative commonly used in meat products, demonstrating that the extract can be as or more effective than sodium nitrite. This finding is mainly due to the presence of the pigment betalain and other phenolic compounds in the fruit.

When incorporating 2.5% of *Opuntia* extract in salami, replacing sodium nitrite preservative and the cochineal carmine dye, Kharrat et al. [61] obtained an improvement in the microbiological stability of products and in the water retention capacity without causing sensory and texture changes. The microorganisms surveyed were mesophiles, molds and yeasts, *S. aureus*, *Clostridium perfringens* and *Salmonella*, and all counts were within limits established by the legislation for both the control treatment and salami with added natural extract, concluding that this is a good alternative for maintaining shelf life during cold storage of this type of meat product.

Prickly pear and other *Opuntia* species, although showing an impressive profile of bioactive compounds, are not well valued in the country and in other parts of the world. Therefore, research on the antimicrobial capacity and the application of extracts from this fruit in meat products can be a good option for offering safe and cleaner label products.

## 4. Conclusions

The data shown in the present study reinforce the possibility of substituting, or at least reducing, the use of synthetic preservatives with natural versions, such as those from fruits and agro-industrial waste, such as peels and seeds. In addition to reducing the environmental impact through the reuse of residues that would initially be discarded, the use of preservatives from natural sources meets the demand of consumers who are increasingly concerned with the possible relationship between the indiscriminate use of synthetic additives and the incidence of health problems. Therefore, the search for plant extracts with antimicrobial activity is an area of study on the rise and there are several reports in the literature on natural alternatives, both for application in meat products and in other food classes, which, therefore, generates a challenge regarding the use of these ingredients in concentrations that are significantly effective against microbiological proliferation, while maintaining the sensory and technological properties of products without compromising their safety.

## Figures and Tables

**Table 1:** Minimum inhibitory concentration in vitro of grape extracts on bacteria.

Strain	Gram	MIC	Grape Cultivar	Reference
*Staphylococcus aureus*	+	6.25 g/L	Pinot Noir *(V. vinifera)*	Martin et al. [24]
		1.56 g/L	Petit Verdot *(V. vinifera)*	Martin et al. [24]
		100.0 mL/L	Tempranillo *(V. vinifera)*	Adámez et al. [37]
*Listeria innocua*	+	100.0 mL/L	Tempranillo *(V. vinifera)*	Adámez et al. [37]
*Listeria monocytogenes*	+	12.5 g/L	Pinot Noir *(V. vinifera)*	Martin et al. [24]
		6.25 g/L	Petit Verdot *(V. vinifera)*	Martin et al. [24]

MIC: minimal inhibitory concentration.

**Table 2:** Minimum inhibitory concentration and minimum bactericidal concentration in vitro of *Opuntia* extract and sodium nitrite on bacteria.

Strain	Gram	MIC (mg/L)		MBC (mg/L)	
		OE	E250	OE	E250
*Bacillus cereus*	+	62.5	125.0	500.0	500.0
*Staphylococcus aureus*	+	62.5	62.5	250.0	500.0
*Escherichia coli*	-	125.0	250.0	500.0	>1000.0
*Salmonella enteric*	-	125.0	500.0	1000.0	1000.0

MIC: minimal inhibitory concentration; MBC: minimal bactericidal concentration; OE: *Opuntia* extract; E250: sodium nitrite. Adapted from Kharrat et al. [62].
